# Remote cardiac rehabilitation program during the COVID-19 pandemic for patients with stable coronary artery disease after percutaneous coronary intervention: a prospective cohort study

**DOI:** 10.1186/s13102-023-00688-2

**Published:** 2023-07-06

**Authors:** Junjie Gu, Xiaoshan Tong, Shasha Meng, Shuhui Xu, Jinyan Huang

**Affiliations:** 1 Department of Cardiology, Hangzhou First People’s Hospital, Zhejiang University School of Medicine, No. 261, Huansha Road, Shangcheng District, Hangzhou, 310006 Zhejiang China; 2 Operation Room, Hangzhou Women’s Hospital, Hangzhou, 310016 Zhejiang China

**Keywords:** Coronary artery disease, Percutaneous coronary intervention, Remote cardiac rehabilitation, Exercise capacity, Mental health

## Abstract

**Objective:**

The coronavirus disease-19 (COVID-19) pandemic restricts rapid implementation of in-person delivery of cardiac rehabilitation (CR) at the center for coronary artery disease (CAD) patients undergoing percutaneous coronary intervention (PCI), thus enabling a cohort comparison of in-person vs. remote CR program. This study aims to investigate outcomes of exercise capacity, health-related quality of life (HRQL), mental health, and family burden of stable CAD patients undergoing PCI in low-to-moderate risk after different delivery models of CR program.

**Methods:**

The study included a cohort of stable CAD patients undergoing PCI who had experienced two naturally occurring modes of CR program after hospital discharge at two time periods, January 2019 to December 2019 (in-person CR program) and May 2020 to May 2021 (remote CR program). The exercise capacity was assessed by means of 6-min walk test (6MWT), maximal oxygen uptake (VO_2_max) and the respiratory anaerobic threshold (VO_2AT_) before discharge, at the end of the 8-week and 12-week in-person or remote CR program after discharge.

**Results:**

No adverse events occurred during the CR period. CAD patients had a longer distance walked in 6 min with a higher VO_2max_ after 8-week and 12-week CR program whether in-person or remote model (p < 0.05). The distance walked in 6 min was longer and the maximal oxygen uptake (VO_2_max) was higher at the end of the 12-week in-person or remote CR program than 8-week in-person or remote CR program (p < 0.05). The respiratory anaerobic threshold (VO_2AT_) of CAD patients was decreased after 8-week CR program whether in-person or remote model (p < 0.05). CAD patients receiving remote CR program exhibited higher HRQL scores in domains of vitality (p = 0.048), role emotional (p = 0.039), mental health (p = 0.014), and the summary score of the mental composite (p = 0.048) compared to in-person CR program after 8 weeks. The anxiety and depression scores of CAD patients undergoing PCI were decreased after 8-week CR program whether in-person or remote model (p < 0.05). The CAD patients receiving remote delivery showed lower anxiety and depression scores compared to those receiving in-person delivery at the end of the 8-week CR program (p < 0.05). It was found that the family burden scores of CAD patients undergoing PCI were reduced after 8-week and 12-week CR program whether in-person or remote model (p < 0.05). The CAD patients receiving remote CR program showed lower family burden scores than those receiving in-person CR program after whether 8 weeks or 12 weeks (p < 0.05).

**Conclusion:**

These data indicate that a properly designed and monitored remote delivery represents a feasible and safe model for low-to-moderate-risk, stable CAD patients undergoing PCI inaccessible to in-person CR during the COVID-19 pandemic.

## Introduction

Coronary artery disease (CAD) is one of the most common cardiovascular diseases and becomes the major cause of morbidity and mortality worldwide until 2040 [1, 2]. Percutaneous coronary intervention (PCI) is a surgical procedure involving the combination of coronary angioplasty with stenting and used to open clogged or narrow coronary arteries of the heart found in CAD [3]. Although effective preventive medication is a priority, patients with stable CAD commonly undergo revascularization by virtue of PCI to reduce symptoms and prevent adverse events [4]. Of note, CAD patients undergoing PCI are required to receive cardiac rehabilitation (CR) programs to optimize lifestyle, restore or increase physical abilities, and reduce major adverse cardiac events [5]. Physical exercise represents a predominant part of CR and can improve prognosis and quality of life of CAD patients undergoing PCI [6]. CR delivers a cost-effective and structured exercise, education, and risk reduction programme, which could contribute to an improvement of 5% of predicted fitness associated with a corresponding 10% reduced risk of cardiac hospitalization or all-cause mortality [7]. Despite these benefits and recommendations in clinical practice guidelines, CR programs are available to only 38.8% of countries [8]. Accordingly, exercise-based CR is highly recommended by all international guidelines for stable CAD patients after hospitalization in hospitals or outpatient centers.

On January 30, 2020, the World Health Organization declared the outbreak of a new Corona virus, SARS-CoV-2 which spread very rapidly throughout the world and impinged on economic, social, and health systems [9]. The sustained coronavirus disease 2019 (COVID-19) pandemic leads to the saturation of hospital services, and rehabilitation centers in many institutes or specialized CR clinics had to close [10]. The COVID-19 infection may contribute to a poor prognosis for stable CAD patients undergoing PCI or CABG, which has raised challenges and dilemmas to their functional recovery, even leading to severe cardiovascular events and deaths [11]. The CAD patients, especially those older patients, may be more likely to infect SARS-CoV-2 virus in their travels to and assembly in hospital services and rehabilitation centers [12]. Therefore, it is urgent to find an alternative CR delivery model to break these barriers, including COVID-19 quarantine, stay-home orders, and recreation facility closures in the local community and reduce the risk of COVID-19 infection for stable CAD patients undergoing PCI and requested to receive CR program. Remote delivery of CR program has been adapted from the existing center-based CR program to expand coverage and accessibility to these programs without increasing total costs [13]. This CR delivery model consists of remote supervision on exercise training and group or individual education meetings for cardiac prevention and management heart disease by videoconference [14]. Importantly, a previous systematic review demonstrated a very low risk of the incidence of adverse events during remote CR program and encourage cardiac patients to be more active in their environment and practice physical exercise regularly [15]. This study aims to investigate outcomes of exercise capacity, quality of life, mental health, and family burden of stable CAD patients with low-to-moderate risk by a cohort comparison of in-person vs. remote CR program.

## Materials and methods

### Participants and eligibility

The study included a cohort of stable CAD patients undergoing PCI who had experienced two naturally occurring modes of CR program after hospital discharge at two time periods, January 2019 to December 2019 (in-person CR program) and May 2020 to May 2021 (remote CR program). To ensure individualized exercise prescription, the patients were given a comprehensive assessment for CR program during the 2–4 weeks after hospital discharge, including review of medical history, risk factor profiling, exercise and lifestyle behaviors, anxiety and depression evaluation, and digital health literacy [16]. Afterwards, each patient was given 12-week in-person or remote CR program.

Participants were deemed eligible if they fulfilled the following inclusion criteria: an age of ≤ 75 years; a cardiologist-confirmed final diagnosis of stable CAD with low-to-moderate risk (< 15% five-year risk of major adverse cardiovascular event) according to the clinical practice guidelines updated 2019 of the American College of Cardiology/American Heart Association Task Force [17]; undergoing PCI with drug-eluting stent within the last 6 weeks, and undergoing completed ambulatory CR program for at least over 5 weeks without adverse cardiac events. In addition to the above inclusion criteria, patients who had received remote CR program must have access to internet and prepare an indoor exercise bike, and have family members or caregiver to support remote CR.

Participants were excluded from the study if they had high risk of CR, recent onset of acute myocardial infarctions within three weeks, malignant arrhythmias, uncontrolled ventricular rhythm disorders, unstable angina, poorly controlled hypertension, exercise-induced ischemia, associated valvular heart disease, COVID-19 vaccination, or infected with COVID-19. Pacemakers or implantable cardioverter defibrillator and cardiac resynchronization therapy (CRT-ICD) carriers, those with severe cognitive impairment or lack of digital literacy, and those failing to walk for long periods of time due to respiratory or musculoskeletal conditions were also excluded.

### In-person CR program protocols

**Fig. 1:**
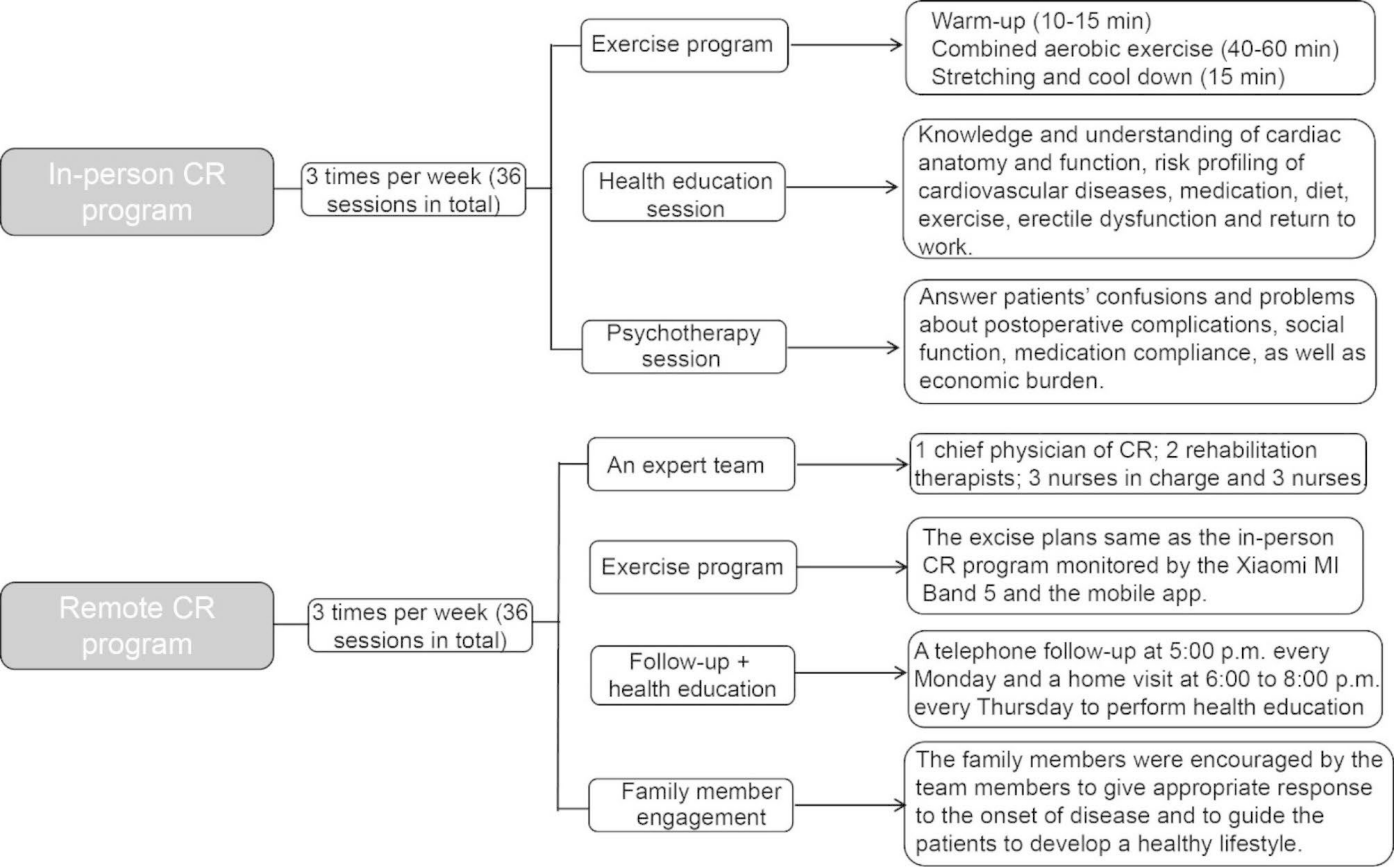
Experimental design of two CR protocols

Patients who were required to receive in-person CR program attended the CR center of our hospital three times per week (36 sessions in total) to perform the exercise program including 10–15 min of warm-up, 40–60 min of combined aerobic exercise (10–20 min of lower body ergometer exercise, 20 min of walking on a treadmill, and 10 min of upper body ergometer exercise) at 50-80% of their peak heart rate, and a final 15 min of stretching and cool down. In addition to physical training, the patients were also required to receive a health education session and a weekly group psychotherapy session, once a week for each, at the hospital. The aim of a health education session was to extend patients’ knowledge and understanding of cardiac anatomy and function, risk profiling of cardiovascular diseases, medication, diet, exercise, erectile dysfunction and return to work. A group psychotherapy session was carried out to handle patients’ confusions and problems about postoperative complications, social function, medication compliance, as well as economic burden. The in-person CR protocols are described in Fig. 1.

### Remote CR program protocols

An expert team specific for implementing the remote CR program was established including a chief physician of CR as a leader, 2 rehabilitation therapists responsible for individualized exercise plans and exercise intensity, 3 nurses in charge, and 3 nurses responsible for health education, psychotherapy session, and data collection via a telephone follow-up every Monday and a home visit every Thursday. Besides, the Xiaomi MI Band 5 (Xiaomi, Beijing, China) was used as a rehabilitation tool to monitor patients’ heart rate (HR) during exercise and the fitness data of patients can be collected by the mobile app named Xiaomi Sports issued by Xiaomi. In brief, patients who were required to receive remote CR program were instructed to connect their smartphones with the Xiaomi MI Band 5 via the mobile app recording the basic information of patients, including age, height, body mass index (BMI), and exercise plans before discharge. Before home exercise, the wristband app and HR sensor were checked and validated with the aid of the family member and the nursing staff. The patients were requested to do home exercise every two days per week and the excise plans were same as in-person CR program, although they were encouraged to exercise every day. When taking exercise, the patients should wear the Xiaomi band on their wrists and be accompanied by their family members. Before discharge, the heart rate, blood pressure, and metabolic equivalent monitored by the wristband app and HR sensor should be adjusted with the data measured by cardiopulmonary exercise data at the hospital to ensure the accuracy of the Xiaomi band detection. The patients’ heart rate, blood pressure, metabolic equivalent and records of movement track were monitored by the Xiaomi band in real time, and the corresponding data were transmitted to the mobile phone application through Bluetooth intelligent technology during movement. If arrhythmia occurs on the electrocardiogram during exercise, the system will remind the patient to stop exercise, and upload the data to the data center in real time. The expert team group will analyze the data and adjust the exercise plan according to the change of the patient’s heart rate and physical condition during exercise. In addition to home exercise, the members in the expert team group performed a telephone follow-up at 5:00 p.m. every Monday and a home visit at 6:00 to 8:00 p.m. every Thursday. The patients’ medication, diet, mental health, and exercise plan were recorded. During each visit, the team members performed health education, addressed the patients’ doubts or problems from the following four aspects: postoperative complications, economic burden, social function limitation and medication compliance, and helped them to manipulate a detailed and feasible solution. At the same time, the family members were encouraged by the team members to give appropriate response to the onset of disease and to guide the patients to develop a healthy lifestyle. The remote CR program was also performed three times per week (36 sessions in total). The remote CR protocols are described in Fig. 1.

### Exercise capacity

The exercise capacity was assessed by means of 6-min walk test (6MWT), maximal oxygen uptake (VO_2_max) and the respiratory anaerobic threshold (VO_2AT_) before discharge, after 8-week CR program, and after 12-week CR program. The 6MWT was conducted following the previously standard guidelines [18] without practice. The patients were instructed to walk 44 m in length continuously as they could on a hospital corridor. The total distance each patient walked in 6 min was recorded. Considering the effects of motivation and perceived exertion of the subject on VO_2_max, VO_2AT_ measurement was also performed for objectiveness and good reproducibility [19]. The cardiopulmonary exercise testing (CPET) applied to determine VO_2_max and VO_2AT_.

### Health-related quality of life (HRQL)

The HRQL of patients applied the short-form health survey (SF-36) which comprises 36 items in total covering the physical composite scores including physical functioning, role physical, bodily pain, general health, and the mental composite scores including vitality, social functioning, role emotional, and mental health [20]. The scales range from 0 to 100, with a higher score reflecting a better HRQL when the score is over 50. The value of Cronbach’s α was 0.846.

### Assessment of anxiety and depression

Assessment of anxiety symptoms and depressive disorders applied the Chinese versions of the Zung’s Self-Rating Anxiety Scale (SAS) and Self-Rating Depression Scale (SDS) both of which cover psychological and somatic symptoms and can intuitively reflect respondents’ subjective feelings for nearly a week [21]. Each scale consisted of 20 items and each item was scored from 1 to 4 (never or very infrequently to most or all of the time). The raw scores in total range from 20 to 80. A score < 50 reflects absence of depression, a score between 50 and 59 reflects mild depression, a score between 60 and 69 reflects moderate depression, and a score > 70 reflects severe depression. The value of Cronbach’s α was 0.824.

### Assessment of family burden

Assessment of family burden applied a Chinese version of the family burden scale of disease (FBS) which encompasses 24 items referring to six domains: economic burden, impact on daily activities, impact on social life, impact on free time, impact on physical health, and impact on mental health [22]. Each item was scored from 0 to 2, with a higher score indicative of a greater burden. The value of Cronbach’s α was 0.792.

### Statistical analysis

A sample of 80 patients was required for the study after sample size power analysis using the PASS software, with a of 0.05, power (1-β) of 80%, and predicted loss rate of follow-up = 10%. The outcomes were described using mean ± standard deviation for measurement variables, using counts and percentages for categorical variables. Fisher’s exact test and Student’s t-test were used to compare the baseline characteristics of the groups, and the repeated measures analysis of variance was used to compare before discharge, after 8-week CR program, and after 12-week CR program. The significant difference was set at p < 0.05 for all tests. Descriptive analyses were performed by GraphPad prism 8.0 (GraphPad Software, San Diego, CA, USA).

## Results

### Participant characteristics

**Fig. 2:**
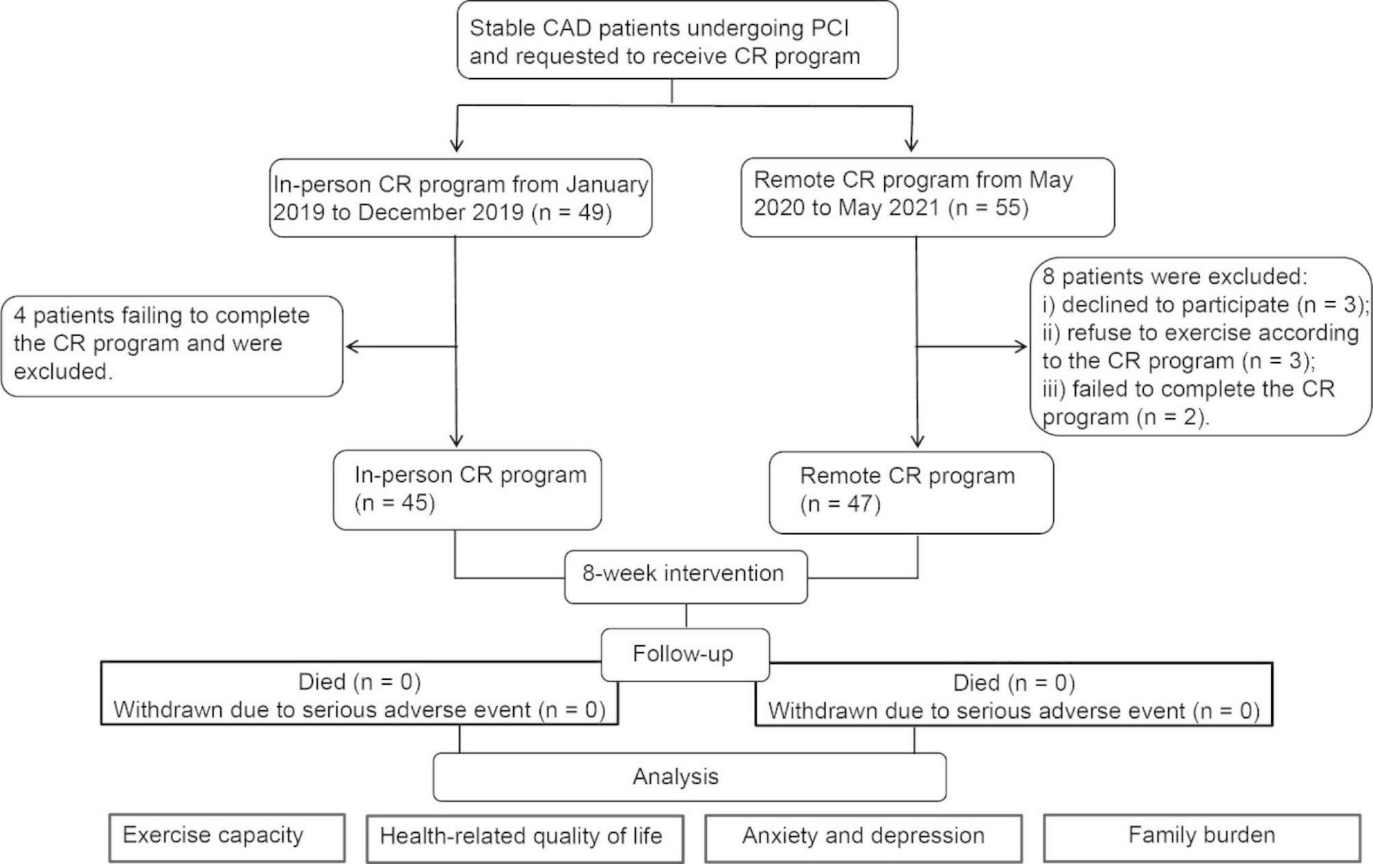
The flow diagram of patient recruitment

A total of 49 stable CAD patients undergoing PCI and requested to receive CR program from January 2019 to December 2019 were prospectively recruited, among which 4 patients failed to completed the in-person CR program for unexplained reasons. Finally, 45 patients (91.84%) completed the in-person CR program were included into this analysis as the in-person CR group. From May 2020 to May 2021, due to the COVID-19 pandemic, 55 patients were requested to receive remote CR program and prospectively invited to participate in the study as the remote CR group. Among 55 patients, 3 patients declined to participate in the study, 2 patients refused owing to their own habits of exercise, and 3 patients failed to complete the remote CR program and withdrew due to difficulty in use of the equipment and other personal reasons. Finally, 47 patients (85.45%) complete the exercise intervention and were included into this analysis as the remote CR group. Figure 2 presents a flow diagram of patient recruitment. No adverse events occurred during the CR period. Table 1 summarized the baseline clinical and demographic characteristics of both groups, including age, sex, education level, the proportions of hypertension and diabetes, BMI, the proportion of current smokers, the number of diseased vessels, the number of stents, total stent length, and hospital length of stay (LOS). No significant differences were noted at baseline between the in-person CR and remote CR groups (p > 0.05).

**Table 1:** Participant characteristics

Item	In-person CR (n = 45)	Remote CR (n = 47)	t/χ^2^	*p*
Age	63.04 ± 10.44	63.49 ± 9.53	0.218	0.828
Sex (male%)	26 (57.78%)	32 (61.70%)	1.024	0.306
Education level				
Primary school and below	11 (24.45%)	12 (25.53%)	0.120	0.904
Middle or high school	20 (44.44%)	25 (53.19%)	1.043	0.297
College and above	14 (31.11%)	10 (21.28%)	1.074	0.283
Hypertension and diabetes (%)	25 (55.56%)	29 (61.70%)	0.134	0.672
BMI (kg/m^2^)	29.01 ± 3.48	28.47 ± 3.67	0.724	0.472
Current smokers (%)	5 (11.11%)	4 (8.51%)	0.420	0.675
Number of diseased vessels (%)				
One-vessel disease	23 (51.11%)	25 (53.19%)	0.200	0.842
Two-vessel disease	12 (26.67%)	14 (29.79%)	0.332	0.740
Three-vessel disease	6 (13.33%)	6 (12.77%)	0.081	0.937
Left main disease	4 (8.89%)	2 (4.25%)	0.900	0.368
Number of stents				
One	23 (51.12%)	25 (53.19%)	0.200	0.842
Two	20 (44.44%)	18 (38.30%)	0.599	0.550
Three	2 (4.44%)	4 (8.51%)	0.790	0.430
Total stent length (mm)	37.28 ± 22.11	36.98 ± 21.79	0.066	0.948
Hospital LOS (d)	4.87 ± 4.63	4.18 ± 5.29	0.665	0.508

CR, cardiac rehabilitation; BMI, body mass index; LOS, length of stay

### The exercise capacity of stable CAD patients undergoing PCI after in-person CR and remote CR models

Before undergoing either in-person or remote CR program, two groups of stable CAD patients did not differ with regard to 6MWT, VO_2max_ and VO_2AT_ at the baseline (p > 0.05). As shown in Table 2, the patients had a longer distance walked in 6 min after undergoing 8-week and 12-week CR program whether in-person or remote model (p < 0.05), and the distance walked in 6 min by the patients also showed significant difference between 8-week and 12-week CR programs (p < 0.05). There was no notable difference in term of the distance walked in 6 min by the patients between in-person and remote CR program after whether 8 weeks or 12 weeks (p > 0.05). Similarly, the VO_2max_ of the patients was enhanced after 8-week and 12-week CR program whether in-person or remote model, and the VO_2max_ was increased at the end of 12-week CR program compared to 8-week CR program (p < 0.05). The VO_2AT_ of CAD patients was decreased after 8-week CR program whether in-person or remote model (p < 0.05). These data indicated that in-person CR program could be replaced by remote CR program for stable CAD patients undergoing PCI during the COVID-19 pandemic.

**Table 2:** Exercise capacity of stable CAD patients undergoing PCI after in-person CR and remote CR models

Outcome	CR program model	Baseline	After 8 weeks	After 12 weeks
6MWT	In-person (n = 45)	456.37 ± 18.09^*#^	534.69 ± 19.29^#^	595.13 ± 23.20
	Remote (n = 47)	461.83 ± 17.20^*#^	541.33 ± 20.04^#^	601.52 ± 22.17
	*p*	ns	ns	ns
VO_2_max	In-person (n = 45)	19.89 ± 1.27^*#^	21.40 ± 1.21^#^	23.09 ± 1.18
	Remote (n = 47)	19.66 ± 1.24^*#^	21.77 ± 1.35^#^	23.35 ± 1.11
	*p*	ns	ns	ns
VO_2AT_	In-person (n = 45)	13.20 ± 0.50^*^	12.95 ± 0.89^#^	13.29 ± 0.63
	Remote (n = 47)	13.32 ± 0.63^*^	13.14 ± 0.70^#^	13.26 ± 0.60
	*p*	ns	ns	ns

CR, cardiac rehabilitation; 6MWT, 6-min walk test; VO_2_max, maximal oxygen uptake; VO_2AT_, respiratory anaerobic threshold; * p < 0.05 compared to 8-week CR program; # p < 0.05 compared to 12-week CR program; ns, not significant

### The HRQL of stable CAD patients undergoing PCI after in-person CR and remote CR models

As depicted in Table 3, following 8-week and 12-week CR program whether in-person or remote model, the HRQL scores were increased in all domains of physical and mental composites both (p < 0.05). The patients receiving remote CR program exhibited higher HRQL scores in domains of vitality (p = 0.048), role emotional (p = 0.039), mental health (p = 0.014), and the summary score of the mental composite (p = 0.048) compared to in-person CR program after 8 weeks, suggesting that stable CAD patients undergoing PCI requested to receive CR program may benefit more from remote delivery especially on mental health during the COVID-19 pandemic.

**Table 3:** The HRQL of stable CAD patients undergoing PCI after in-person CR and remote CR models

Outcome	CR program model	Baseline	After 8 weeks	After 12 weeks
Physical functioning	In-person (n = 45)	70.12 ± 18.82^*#^	78.77 ± 17.43	80.51 ± 17.33
	Remote (n = 47)	68.68 ± 16.95^*#^	80.15 ± 20.03	80.83 ± 18.49
	*p*	ns	ns	ns
Role physical	In-person (n = 45)	52.12 ± 44.26^*#^	72.43 ± 46.39	73.21 ± 43.17
	Remote (n = 47)	54.03 ± 43.52^*#^	75.38 ± 44.43	74.60 ± 40.92
	*p*	ns	ns	ns
Bodily pain	In-person (n = 45)	64.12 ± 22.41^*#^	76.63 ± 21.20	74.72 ± 27.10
	Remote (n = 47)	62.70 ± 21.52^*#^	74.83 ± 20.44	74.16 ± 21.06
	*p*	ns	ns	ns
General health	In-person (n = 45)	48.73 ± 18.17^*#^	55.75 ± 20.12	58.56 ± 22.43
	Remote (n = 47)	46.45 ± 19.03^*#^	54.04 ± 15.46	52.82 ± 20.69
	*p*	ns	ns	ns
Physical composite	In-person (n = 45)	58.77 ± 23.60^*#^	70.90 ± 19.47	71.75 ± 21.69
	Remote (n = 47)	57.97 ± 21.41^*#^	71.10 ± 20.55	70.60 ± 23.05
	*p*	ns	ns	ns
Vitality	In-person (n = 45)	52.13 ± 22.58^*#^	62.05 ± 19.26	63.44 ± 19.66
	Remote (n = 47)	53.35 ± 20.20^*#^	69.86 ± 18.04	65.27 ± 25.63
	*p*	ns	**0.048**	ns
Social functioning	In-person (n = 45)	57.53 ± 23.64^*#^	87.35 ± 15.40	86.69 ± 20.31
	Remote (n = 47)	58.82 ± 25.04^*#^	86.80 ± 17.14	86.06 ± 19.20
	*p*	ns	ns	ns
Role emotional	In-person (n = 45)	70.45 ± 30.56^*#^	80.19 ± 27.12	87.56 ± 30.12
	Remote (n = 47)	72.08 ± 31.11^*#^	91.84 ± 26.14	88.57 ± 28.93
	*p*	ns	**0.039**	ns
Mental health	In-person (n = 45)	68.28 ± 12.10^*#^	74.45 ± 13.37	77.44 ± 18.13
	Remote (n = 47)	70.57 ± 12.84^*#^	81.96 ± 15.26	79.69 ± 16.44
	*p*	ns	**0.014**	ns
Mental composite	In-person (n = 45)	63.10 ± 21.55^*#^	76.01 ± 14.36	78.78 ± 21.19
	Remote (n = 47)	63.71 ± 20.77^*#^	82.62 ± 17.10	79.90 ± 20.47
	*p*	ns	**0.048**	ns

* p < 0.05 compared to 8-week CR program; # p < 0.05 compared to 12-week CR program; ns, not significant

### Anxiety symptoms and depressive disorders in stable CAD patients undergoing PCI after in-person CR and remote CR models

Next, we analyzed the anxiety symptoms and depressive disorders of stable CAD patients undergoing PCI after in-person CR and remote CR models by using Zung’s SAS and SDS questionnaires. As listed in Table 4, the SAS and SDS scores of patients were decreased after 8-week CR program whether in-person or remote model (p < 0.05). The patients receiving remote CR program showed lower SAS and SDS scores compared to those receiving in-person CR program for 8 weeks (*p* < 0.05). These data suggest that remote CR program could effectively alleviate the depression and anxiety of patients requested to receive CR program during the COVID-19 pandemic.

**Table 4:** The anxiety symptoms and depressive disorders of stable CAD patients undergoing PCI after in-person CR and remote CR models

Outcome	CR program model	Baseline	After 8 weeks	After 12 weeks
SAS	In-person (n = 45)	9.32 ± 1.42^*#^	8.65 ± 1.30	8.63 ± 1.32
	Remote (n = 47)	9.29 ± 1.45^*#^	8.03 ± 1.18	8.16 ± 1.24
	*p*	ns	**0.019**	ns
SDS	In-person (n = 45)	8.80 ± 1.45^*#^	8.23 ± 1.20	8.12 ± 1.19
	Remote (n = 47)	8.69 ± 1.50^*#^	7.70 ± 1.10	8.07 ± 1.18
	*p*	ns	**0.030**	ns

SAS, Self-Rating Anxiety Scale; SDS, Self-Rating Depression Scale; * p < 0.05 compared to 8-week CR program; # p < 0.05 compared to 12-week CR program; ns, not significant

### Family burden of stable CAD patients undergoing PCI after in-person CR and remote CR models

The family burden of stable CAD patients undergoing PCI after in-person CR and remote CR models was assessed by using the FBS scale (Table 5). It was found that the FBS scores of patients were reduced after 8-week and 12-week CR program whether in-person or remote model (p < 0.05). The patients receiving remote CR program showed lower FBS scores than those receiving in-person CR program after whether 8 weeks or 12 weeks (*p* < 0.05). These data suggest that stable CAD patients undergoing PCI requested to receive CR program may cause less family burden during the COVID-19 pandemic.

**Table 5:** The FBS scores of stable CAD patients undergoing PCI after in-person CR and remote CR models

Outcome	CR program model	Baseline	After 8 weeks	After 12 weeks
FBS	In-person (n = 45)	23.09 ± 9.32^*#^	21.02 ± 8.04	23.39 ± 9.12
	Remote (n = 47)	23.02 ± 8.68^*#^	17.24 ± 7.24	18.36 ± 7.18
	*p*	ns	**0.020**	**0.004**

FBS, family burden scale of disease; * p <  0.05 compared to 8-week CR program; # p < 0.05 compared to 12-week CR program; ns, not significant

## Discussion

This study examined the exercise capacity, quality of life, mental health, and family burden of stable CAD patients undergoing PCI requested to receive in-person CR program compared to remote CR program. The main findings of this work support remote delivery as a feasible and safe model for stable CAD patients undergoing PCI inaccessible to in-person CR during the COVID-19 pandemic.

Exercise capacity is considered as a predictor of cardiovascular death in patients with CAD [23, 24]. Some stable CAD patients undergoing PCI are afraid of stent detachment due to exercise. Over time, their cardiac function gradually weakened, which has a serious impact on the long-term efficacy of patients. Exercise can help to maintain blood pressure control, improve cardiorespiratory fitness, enhance the function of vascular endothelial cells, and prevent the development of atherosclerosis [25]. Similar to our study, Candelaria et al. analyzed the patient experience while exercising between in-person and remote-delivered CR exercise interventions during the COVID-19 pandemic [26]. Although they thought remote-delivered CR program during the COVID-19 pandemic had equivalent patient experience, sometimes better HRQL outcomes than in-person model, the rapid transition from in-person to remote delivery in the same group of patients may contribute to less careful planning and testing of remote interventions, creating a further cross-sectional study. In the study reported by Batalik et al., they endorsed the feasibility of remote delivery as an alternative delivery model for CR program, focusing on 200 m fast walking test after 8-week intervention to reflect exercise capacity of CAD patients [27]. However, only 19 participants and the absence of a control group in the above study means a further investigation in a larger-scale comparative study during the COVID-19 pandemic. Montoye et al., also demonstrated improved mental health and physical fitness by virtual exercise program during the COVID-19 pandemic for adults with chronic disease [28]. In our study, we compared the exercise capacity results between 45 patients completing the in-person CR program and 47 patients completing the exercise intervention by remote delivery. The data showed that in-person delivery of CR program and remote delivery of CR program both can improve the exercise capacity of stable CAD patients undergoing PCI and their exercise capacity did not differ between in-person delivery of CR program or remote delivery of CR program.

Following 8-week and 12-week CR program whether in-person or remote model, the HRQL scores were increased in all domains of physical and mental composites both, indicating the overall equivalence in HRQL between groups was expected, concurring with other previous reports [29, 30]. Similar have also been noted in other conditions such diabetes, and remote monitoring of physiological, symptom, and self-care behavior data did not improve or have a deleterious effect on quality of life or psychological outcomes compared to those did not receive remote model [31]. CR is a complex intervention with many interacting elements. It is therefore difficult to pinpoint the active programme components, which could also differ for each patient’s perspective. Specifically, in our study, the patients receiving remote CR program exhibited higher HRQL scores in domains of the mental composite compared to in-person CR program after 8 weeks, suggesting that CAD patients undergoing PCI requested to receive CR program may benefit more from remote delivery especially on mental health during the COVID-19 pandemic. The additional benefits for mental health in remote CR program may have been a result from pandemic-related isolation distress [32], and thus remote delivery for CR program may be an effective and timely intervention as it was individualized. During the remote delivery for CR program, patients have one-on-one contacts to raise specific recovery concerns and receive more personalized counselling and motivational support than in-person delivery [33]. The patients receiving in-person CR exercise could engage with the nursing staff through incidental conversations and questions and obtain encouragement from the exercise professionals during exercise. For remote-delivery CR participants, they appreciated remote delivery due to easy accessibility from the home, less travel time, direct engagement and encouragement from their lives. Individualized and personal contacts with cardiovascular specialists regularly in the remote CR program make participants feel more flexible and less stressed than in the in-person CR program. As previously reported by Su et al., actionable CR guidance, increased awareness, and professional support in early post-discharge consultation could significantly reassure patients [34]. Therefore, remote delivered CR program was demonstrated to be a suitable alternative model for patients who are unable to participate in the in-person program, particularly as other studies have demonstrated that low-risk patients could be safely managed without requiring ongoing biochemical evaluations [35].

For stable CAD patients undergoing PCI, depression and anxiety are associated not only with a poorer prognosis and increased long-term mortality, but also with reduced productivity owing to evidently increased disability rates in the working population and increased medical care costs [36]. After PCI, fear of disease recurrence, unknown prognosis and high medical costs will increase psychological burden and induce depression in stable CAD patients undergoing PCI. Anxiety and depression are independent risk factors in the pathophysiological progress of CAD, which run through the whole process of disease treatment, rehabilitation and prevention, and increase the risk of patient death [37]. In this study, remote CR program was as effective as the in-person CR program in improving anxiety and depression symptoms, functional capacity, and quality of life. More specifically, the patients receiving remote CR program showed lower SAS and SDS scores compared to those receiving in-person CR program for 8 weeks. Exercise together with educational and psychological interventions in remote CR program had a positive impact on the psychological state of the patients.

After PCI, patients suffered from in stent restenosis repeatedly, and the high medical costs brought a heavy economic burden to patients’ families. The economic burden has a negative impact on the physiological health and emotional functions of the caregivers, especially during the COVID-19 pandemic [38]. Remote delivered CR program in the home environment represent an opportunity to increase overall utilization as most evidence found strong evidence that remote CR program was cost-effective [13]. Scherrenberg et al. analyzed the effectiveness of a home-based mobile CR program in elderly patients that were unable or not willing to receive center-based CR from a health-economic point of view, demonstrating the home-based mobile CR program was cost-effective alternative to improve cardiorespiratory fitness [39]. In addition, due to the illness of patients after PCI, their spouses have more psychological problems due to the disorder of family and marriage life and the lack of sexual life, which leads to greater psychological burden [40, 41]. Through positive psychological suggestion to patients, regular telephone calls and home visits, the remote CR program enables patients and their families to obtain relevant knowledge about diseases at any time outside the hospital, helps patients and their families to maintain an optimistic attitude and positive coping style, instructs their families how to carry out family self-rescue, and improves the preventive intervention ability of caregivers. This service concept is based on continuous nursing, extending the patient-centered service concept to families, so that patients can receive continuous treatment and rehabilitation guidance, and provide seamless nursing services for patients. Development of remote CR program into regular practice not only during the COVID-19 pandemic is the next step to yield the same results as would be conferred by the standard rehabilitation process and to overcome potential staff deficiencies and geographical barriers [42]. Future investigations should focus on how to effectively enhance the adherence of patients and the implementation of alternatives based on smart devices to ensure data reliability.

There are several limitations of this study that should be noted. Although the remote CR program was delivered in a fixed format, there is no fidelity test to ensure that the curriculum was delivered exactly as it should be. In the in-person CR group, the participants voluntarily attended the drop-in center, but their family members did not receive any assistance from the drop-in center, unlike the experimental group families who received family psychoeducation. Thus, the benefit of remote CR program may be due to the potential Hawthorne effects, because services offered to the remote CR group were more intensive. Besides, the patient recruitment occurred at two different time points, January 2019 to December 2019 and May 2020 to May 2021 due to the COVID-19 pandemic, creates a critical need for further prospective studies investigating the outcomes after two delivery models of CR program at the same period to validate the remote delivery as an alternative delivery model for CR program.

In conclusion, our study demonstrates that both in-person or remote CR programs could effectively improve exercise capacity, quality of life, mental health, and reduced the family burden of low-to-moderate-risk, stable CAD patients undergoing PCI who were requested to receive CR program. This investigation showed that CR by a remote delivery might become a relevant alternative to conventional center-based CR. This innovative healthcare delivery method appears to be a feasible, tolerable, safe, cost-effective solution, and is likely to facilitate the continuity of care for people who encounter geographical or social accessibility difficulties. In future research, this study should be replicated with a larger sample size, validation of the curriculum, and a comparable intervention intensity control group. For the outcome assessments, areas such as employment, community functioning, personal empowerment, and sense of purpose should be included rather than merely focusing on symptom management and social functioning.

## Data Availability

All data generated or analyzed during this study are included in this published article.
